# Supplementary material for: Chromothripsis during telomere crisis is independent of NHEJ, and consistent with a replicative origin
Source: Genome Res. 2019 May;29(5):737–49. doi: 10.1101/gr.240705.118 (PMC6499312; doi:10.1101/gr.240705.118)
Supplement: Supplemental Material [file supp_gr.240705.118_Supplemental_file_1.zip › contigs/annotated_contigs/DB113/contig.3.DB113_length_1019_mean_cov_19.7674190383.docx]

**DB113_length_1019_mean_cov_19.7674190383**

```
CTGCCCAAAATGGCGAAACCTCATCTCTCCTAAAAATACAAAAATTAGCCAGGCGTGGTTGTGCACACCTGTAATCCCAGCTACTTGGG
 >chr10:12361921-12362294 - E=2e-212 p=0e+00
AGGCTGAGGCAGGAGAATCACTCGAACTGGGGAGGTATAGGTGGCAGTGAGCCCAGCCCGGGCGAAAAGAGCAAGACTCCATCTCAAAA

ACAAAAACAAAAAAGGAGCAGAGGAGACTCAGAAGTGTTTAGGGAATCGGGAGGAGGCCAGCCTGTTATGGTAAATAACAGGAACGGGA

AAGTAACCAGGGACCTGATCTCCAAGGAGCTTCAATTTTAGATGAAGGACTTTGCATTGTCTAGAATTATCTAGATAACAGGCTTTTGG

ATTGATTGATAATGGGC|ACATAGCAAAATCCCGGCTCTACACAATACAAAAAATTAGCCAGGCATGGTGGTGTATGCCTGTAGTCCCA
                   >chr10:12956285-12956647 - E=1e-199
GCTGCTTGGGAGGCTGAGGCAGGCGAATCGCTTGACCCAGGAGGCGGAGGTTGCAGTGAGCCAAGGACACGCCACTGCACTTCAGCCTG

GGTGACAGAGCAAGACTCTGTCTCAAAAGAAAAAACAAAAGATAAAATCAGAAATGAAAGAGGAGAAATTACACTGATACCGCAGAAAT

ACAAATGACTAAAAAAGACTACCACAGACAATCATATGCCAACAAATTAGATAACTTACAAGAAATGGATAAATTCCTAGACACATACA

ACCTACTATGATTAAGTTATGAGT|T|GTGCTGGGATTACAGGTGTGCGTGAGCCACCATGCCTGGCCTGAGTTGTCTTTTTTTACTTA
                          >chr10:12956706-12956989 + E=3e-158
GTCTAGCTAAAGGTTTGTCAGTTTTGTCTTTTCAAAAAACCAACTCTTTATGTTGCTGATCTTCTGTATTATTTTTCTACTCTCTCATT

TATTATTATTTCTTTCCTTCTAACTTTGGGCTTAGTTTGTTCTTCTTTTTGTAGTTCCTTGAGGTAACACTAGATGTTTACTTGGCAAG

TTCTTTTTTGATGCAGGCATTTATTGTTATAAACTTCTCTCTT
```
